# Iron Oxide Nanoparticle Delivery of Peptides to the Brain: Reversal of Anxiety during Drug Withdrawal

**DOI:** 10.3389/fnins.2017.00608

**Published:** 2017-11-01

**Authors:** Nathan Vinzant, Jamie L. Scholl, Chia-Ming Wu, Trevor Kindle, Ranjit Koodali, Gina L. Forster

**Affiliations:** ^1^Division of Basic Biomedical Sciences, Center for Brain and Behavior Research, Sanford School of Medicine, University of South Dakota, Vermillion, SD, United States; ^2^Department of Chemistry, University of South Dakota, Vermillion, SD, United States

**Keywords:** iron oxide, nanoparticle, blood brain barrier, corticotropin-releasing factor, amphetamine, anxiety

## Abstract

Targeting neuropeptide systems is important for future advancements in treatment of neurological and psychiatric illnesses. However, many of the peptides and their analogs do not cross the blood-brain barrier (BBB) efficiently. Nanoparticles such as iron oxide can cross the BBB, and here we describe a novel method for the conjugation of a peptide antisauvagine-30 (ASV-30) to iron oxide nanoparticles. Previous research has shown that direct infusion of ASV-30 into the brain reduces anxiety-like behavior in animal models via actions on corticotropin releasing factor type 2 (CRF_2_) receptors. Therefore, we tested whether iron oxide+ASV-30 complexes cross the BBB of rats and then determined whether iron oxide+ASV-30 nanoparticles are localized with CRF_2_-expressing neurons. Finally we tested the hypothesis that systemic infusion of iron oxide+ASV-30 can reduce anxiety-like behavior. First we describe the synthesis and demonstrate the stability of iron oxide-peptide nanoparticle complexes. Next, nanoparticles (87.7 μg/kg Fe_2_O_3_) with or without ASV-30 (200 μg/kg, ip) were injected into male rats 30 min prior to transcardial perfusion and brain fixation for immunohistochemical analysis, or before testing on the elevated plus maze (EPM) in an amphetamine withdrawal model of anxiety. Systemically administered iron oxide+ASV-30 particles were present in the brain and associated with neurons, including those that express CRF_2_ receptors, but did not localize with the iron storage protein ferritin. Furthermore, systemic administration of ironoxide+ASV-30 reduced amphetamine withdrawal-induced anxiety without affecting locomotion, suggesting that the anxiolytic effects of ASV-30 were preserved and the bioavailability of ASV-30 was sufficient. The findings demonstrate a novel approach to peptide delivery across the BBB and provide insight as to the neural distribution and efficacy of this nanotechnology.

## Introduction

As our understanding of neuropeptide regulation of neuronal function increases, the need to effectively manipulate neuropeptide levels or receptor activity in the brain is becoming more necessary. Neuropeptidergic systems have been implicated in a variety of neuropsychiatric and neurological conditions including depression, anxiety, addiction, and neurodegenerative disorders (Holmes et al., 2003; Davies et al., 2015; Park et al., 2015; Li et al., 2016; Lindholm et al., 2016; Spencer et al., 2016). Exogenously-administered peptides are suggested to have high therapeutic potential because of their wide safety range, relative potency and selectivity (Lalatsa et al., 2014). However, their molecular weight and hydrophobicity limits the ability of peptides to cross the blood-brain barrier (BBB) (Batrakova et al., 2005; Teixido et al., 2005). While transport mechanisms do exist for some brain-relevant peptides, these transporters appear to be highly saturable and brain bioavailability of systemically administered peptides is typically low (Irwin et al., 1994; Egleton et al., 2000; Lalatsa et al., 2014).

In response to the bioavailability problems of systemically-administered peptides, nanomaterials have been developed to transport peptides across the BBB. For example, functionalized polyethylene glycol (PEG) and PEGylated dendrigraft poly-L-lysines (DGL) nanomaterials have been utilized to deliver therapeutically-relevant peptides to the brain (e.g., Karatas et al., 2009; Lui et al., 2016). These require the nanomaterial to be conjugated to receptors or molecules to take advantage of receptor-mediated transport across the BBB but have the potential to significantly alter transport of endogenous ligands reliant on receptor-mediated transport (e.g., transferrin/iron, leptin, insulin) when nanomaterials are administered in chronic, therapeutic settings (Karatas et al., 2009; Lalatsa et al., 2014; Lui et al., 2016). In light of this, iron oxide nanomaterials that transmigrate through the BBB have potential for delivery of peptides to the brain without altering homeostasis of endogenous compounds (Shubayev et al., 2009; Pilakka-Kanthikeel et al., 2013). Iron oxide nanomaterials in the fully oxidized state (Fe_2_O_3_) are stable, are biocompatible, readily cross the BBB leaving the barrier intact, and can be modified to carry peptide cargo (Shubayev et al., 2009; Minchin and Martin, 2010; Yang, 2010; Jain, 2012). However, to prevent aggregation and improve cellular uptake, iron oxide nanoparticles are subjected to various surface functionalizations, but this treatment can increase cellular iron accumulation and induce cytotoxicity (Pisanic et al., 2007; Rivet et al., 2012; Kim et al., 2013). Therefore, the current study explored the utility of 5 nm Fe_2_O_3_ iron oxide nanoparticles with an aminosilane coating as a carrier system to deliver peptides to the brain. Aminosilane functionalization prevents aggregation, allows for amino acid attachment and is not cytotoxic (Natarajan et al., 2008; Rivet et al., 2012).

Corticotropin releasing factor (CRF) is a neuropeptide commonly implicated in fear and anxiety (Liang et al., 1992; Basso et al., 1999; Radulovic et al., 1999; Kikusui et al., 2000; Takahashi, 2001; Nemeroff, 2004; Bale, 2005; Lukkes et al., 2009; Vuong et al., 2010; Bledsoe et al., 2011). Specifically, activation of the CRF type 2 (CRF_2_) receptor in the dorsal raphe nucleus (dRN) increases serotonin activity in the limbic system (Pernar et al., 2004; Forster et al., 2006, 2008; Lukkes et al., 2009; Scholl et al., 2010) and increases anxiety-like behaviors in animal models of early life stress and amphetamine withdrawal (Vuong et al., 2010; Bledsoe et al., 2011; Reinbold et al., 2014). Similarly, CRF_2_ receptors in the lateral septum appear to mediate anxiety-like behavior (Henry et al., 2006; Reinbold et al., 2014). Due to their effects on anxiety-like behaviors in animal models, CRF_2_ receptors have been identified as a potential important target for anxiolytic drug development (Forster et al., 2012). However, like many neuropeptide receptor ligands, CRF_2_ receptor ligands are peptides with low neurobioavailability if administered systemically.

Antisauvagine-30 (ASV-30) is a peptide antagonist with 1,000–10,000 fold greater selectivity for the CRF_2_ over the CRF_1_ receptor (Higelin et al., 2001). Direct infusion of ASV-30 into the brain of rats reduces anxiety-like behaviors of rats in amphetamine withdrawal (Vuong et al., 2010; Reinbold et al., 2014) and in adult rats exposed to early-life stress (Bledsoe et al., 2011). Therefore, the goals of this study were to develop a method for conjugation of ASV-30 with iron oxide nanoparticles and to test the ability of these nanoparticle complexes to cross the BBB, localize with CRF_2_-expressing neurons in the brain, and reduce anxiety-like behavior in rats undergoing amphetamine withdrawal. We also tested the possibility that iron oxide nanoparticles may be sequestered in nervous tissue by ferritin (Moos and Morgan, 2004; Hare et al., 2013), thus reducing bioavailability.

## Materials and methods

### Nanoparticle synthesis

Iron oxide nanoparticles (Fe_2_O_3_) synthesis was modified from Borcherding et al. (2014) and involved refluxing 8.7 mmol FeCl_3_ solution containing 40 mL methanol and 5 mL water at 80°C for 72 h with drop-wise addition of 17.4 mmol NaOH solution containing 30 mL methanol. Iron oxide precipitate formed in the methanol solution, was washed three times using 14 mL of 50% v/v methanol-acetone and dried in an air oven for overnight at 80°C. Power X-ray diffractometry was used to identify the phase (Fe_2_O_3_) and purity (no other oxide phases were detected) of the resultant nanoparticles. Nanoparticles were characterized by transmission electron microscopy to estimate the particle size and size distribution, demonstrating that the resulting nanoparticles were ~5 ± 1 nm in diameter. Nanoparticles were then conjugated with 3-aminopropyltriethoxysilane (APTES) by refluxing the Fe_2_O_3_ nanoparticles with APTES at a 1:4 molar ratio in methanol for 48 h. The resultant powder was collected and washed with a mixture of acetone and methanol in a 1:1 volume ratio solution. The APTES-coated nanoparticles were dried overnight at 80°C and characterized using Fourier-Transform Infrared Spectroscopy (FT-IR) and Thermogravimetric Analysis (TGA) to confirm the presence of APTES and to estimate the amount of APTES groups. APTES functionalization was performed in order to effectively couple fluorescein isothiocyanate (FITC). 5 mg of APTES-coated iron oxide nanoparticles were dissolved into 0.5 mL, 1 × 10^−5^ molar FITC in artificial cerebrospinal fluid (aCSF). The resulting solution was then sonicated for 10 min. After sonication, the powder was collected and washed in aCSF three times. The resultant Fe_2_O_3_ functionalized nanoparticles were monitored for stability over 48 h using UV-Vis spectrophotometry, and compared to UV-Vis spectrum of a 1 × 10^−4^ molar Fe^3+^ standard solution.

Antisauvagine-30 (ASV-30; Thermo Fisher Scientific, Hampton, NH) was attached to the resultant Fe_2_O_3_ nanoparticles by mixing APTES-coated Fe_2_O_3_ nanoparticles with ASV-30 in a 10:1 molar ratio in a vehicle solution comprised of 10% ethanol and 90% aCSF by volume. ASV-30 has multiple carboxyl (C=O) groups which bind to the amine (-NH_2_) groups of APTES through covalent attachment and form amide bonds. In addition, hydrogen bonding and ionic interactions are also possible between ASV-30 and APTES-Fe_2_O_3_ nanoparticles. The solution was monitored hourly by FT-IR for up to 5 h to confirm the successful attachment of ASV-30 to the APTES-coating of the iron oxide nanoparticles. Finally, the stability of the APTES-coated nanoparticles with ASV-30 was assessed by FT-IR spectroscopy in aCSF.

### Animals

Male Sprague-Dawley rats were bred within the in-house Animal Resource Center at the University of South Dakota. Adult (8–12 weeks old) rats were pair-housed in the same room under a 12 h reversed light cycle, with the dark period running from 10:00 to 22:00. Animals had free access to food and water, and cages were changed twice per week. All animal procedures were approved by and performed in accordance with the University of South Dakota institutional animal care and use committee.

### Distribution of systemically administered nanoparticles in the brain

Iron oxide nanoparticles are known to cross the BBB (Silva, 2008; Minchin and Martin, 2010; Yang, 2010), but it is not known if the conjugation of peptide to the nanoparticles will interfere with their transport kinetics. To address this, male adult rats (*n* = 18) were injected (ip.) with a APTES-coated Fe_2_O_3_ nanoparticle solution (87.7 μg/kg Fe_2_O_3_) tagged with FITC, with or without ASV-30 (200 μg/kg; 100-fold greater than intracerebroventricular infusion; Reinbold et al., 2014). No adverse reactions were observed as a result of this injection. Thirty minutes after injection, rats were anesthetized with sodium pentobarbital (100 mg/kg) and transcardially perfused with 150 mL 0.05 M phosphate-buffered saline (PBS) followed by 250 mL 4% paraformaldehyde (Meyer et al., 2012). Brains were collected and stored in 4% paraformaldehyde at 4°C for 20 h, followed by a series of two PBS washes for 24 h each with gentle agitation at 4°C. Brains were cryoprotected in a 30% sucrose solution followed by storage at −80°C. 40 μm coronal sections were taken using a freezing microtome.

Separate series of 40 μm sections were processed and imaged for either nanoparticle (FITC) with the neuronal marker NeuN, or nanoparticle and CRF_2_ receptors with NeuN, or nanoparticle and ferritin with NeuN. Sections were washed three times using 0.04% goat serum (Jackson ImmunoResearch, West Grove, PA) in PBS for 5 min at room temperature and blocked in 2% goat serum in PBS to reduce non-specific antibody binding. Sections were then incubated in either 1:500 rabbit anti-rat ferritin antibody (GenWay Biotech, San Diego, CA, USA; GWB-792B12) or 1:200 rabbit anti-CRF_2_ receptor antibody (Novus Biologicals, Littleton, CO, USA; NBP1-00768) with 1:750 mouse anti-NeuN antibody (Millipore Corporation, Temecula, CA, USA; MAB377; Barr et al., 2010) for 19 h at 4°C followed by 1 h at room temperature with gentle agitation on a shaker plate. Sections were then washed three times in 0.04% goat serum before being incubated in 1:200 goat anti-mouse Cy3 (Jackson ImmunoResearch; 115-165-003) or 1:200 goat anti-rabbit Cy3 (Jackson ImmunoResearch; 111-165-003) and 1:200 goat anti-mouse AMCA (Jackson ImmunoResearch; 115-155-003) secondary antibodies with gentle agitation for 2 h at room temperature. Sections then underwent three final washes in 0.04% goat serum in PBS and were mounted on subbed slides and cover slipped using Prolong Diamond Antifade Mountant (Life Technologies, Carlsbad, CA, USA). Sections from the lateral septum and dRN were then imaged using an Olympus Fluoview 500 laser scanning confocal microscope (Olympus America, NY, USA) at 60x oil immersion. A Kalman scanning filter was used to exclude background noise not caused by specific antibody binding.

### Anxiolytic effects of iron oxide+ASV-30 nanoparticles

All behavioral testing was conducted in the dark phase of the light cycle and were performed in a dark room illuminated by red lighting. To identify any potential effect of iron oxide nanoparticle administration on behavior in the elevated plus maze (EPM), rats were injected (ip.) with either unconjugated APTES-coated nanoparticles (87.7 μg/kg Fe_2_O_3_) in CSF or CSF alone, (*n* = 11/group). Thirty minutes later, rats were placed in the center of the EPM (Noldus Information Technology, Leesburg, VA) and allowed to explore freely for 5 min, with their behavior recorded using an overhead camera via Mediacruise V.2.24 (Canopus Co. Ltd., Kobe, Japan). Following the test, rats were returned to their home cage and monitored for 3 days to identify any health effects of the nanoparticle injection, of which none were observed. Total distance moved and total time spent in open and closed arms of the EPM were scored by Ethovision XT 5.1 (Noldus Information Technology).

To determine anxiolytic effects of ASV-30 delivered as nanoparticle cargo, we utilized a well-established model of anxiety during amphetamine withdrawal (Barr et al., 2010; Vuong et al., 2010; Tu et al., 2014). A separate group of rats were pretreated with either saline or amphetamine (2.5 mg/kg, ip.) for 2 weeks and then allowed to undergo 2 weeks of withdrawal. Animals were then injected with either unconjugated APTES-coated Fe_2_O_3_ nanoparticles in aCSF (87.7 μg/kg Fe_2_O_3_, ip) or APTES-coated Fe_2_O_3_ nanoparticles+ASV-30 (87.7 μg/kg Fe_2_O_3_ and 200 μg/kg ASV-30, ip) 30 min prior to testing (*n* = 13 per group). Rats were tested on the EPM as described above.

### Statistical analysis

Data collected from the first behavioral study comparing nanoparticle administration to aCSF administration was analyzed using a one-way ANOVA. Data collected in the second behavioral study involving amphetamine withdrawal were analyzed using a two-way ANOVA test with factors of pretreatment and treatment. Significant main effects or interactions were followed by a Student-Newman-Keuls *post-hoc* test for multiple comparisons. All data were analyzed using SigmaPlot 11.0.

## Results

### Characterization of nanoparticle complexes

**Figure 1:**
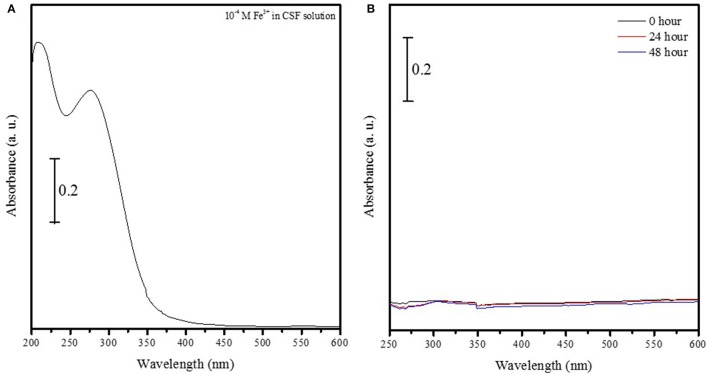
Iron oxide nanoparticles do not dissociate into ionic iron (Fe^3+^ ions) at physiological pH. **(A)** UV-vis spectrum of 1 × 10^−4^ M Fe^3+^ ions in solution shows two strong peaks at 275 and 209 nm. **(B)** UV-vis spectrum of iron oxide nanoparticles conjugated to FITC in aCSF solution do not show characteristic peaks at 275 and 209 nm, thus indicating that nanoparticles do not dissociate into oxygen and ionic iron (Fe^3+^ ions) *in vivo* within either 24 or 48 h.

**Figure 2:**
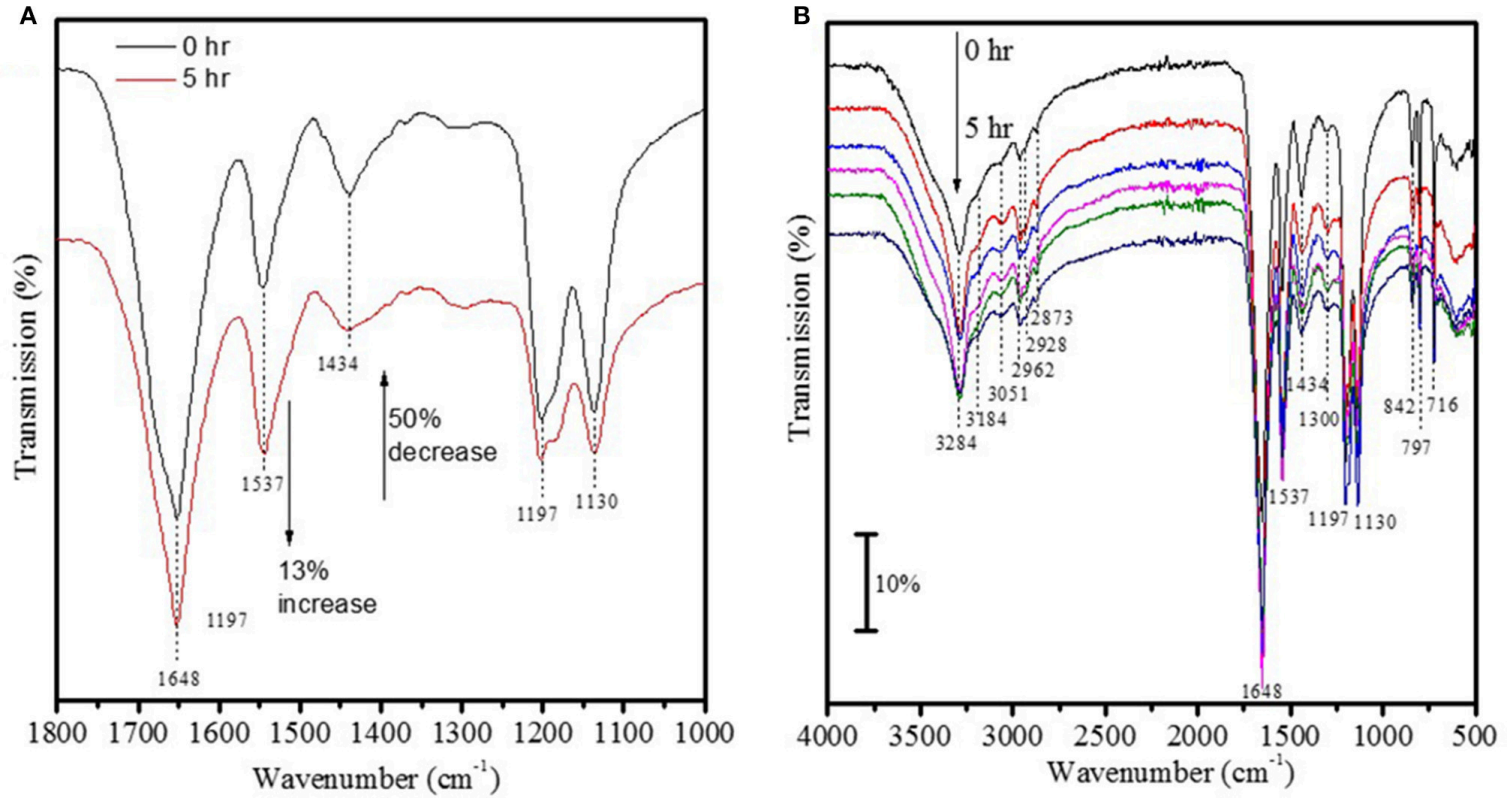
Validation of ASV-30 conjugation with iron oxide surface. **(A)** FT-IR spectra of ASV-30 complexation with APTES-Fe_2_O_3_ coated nanoparticles as a function of time. **(B)** Comparison of FT-IR spectra of ASV-30-APTES-coated Fe_2_O_3_ nanoparticles washed with aCSF to unwashed ASV-30-APTES-Fe_2_O_3_ nanoparticle.

The UV-vis spectrum of 1 × 10^−4^ M Fe^3+^ ions in solution showed two strong peaks at 275 and 209 nm (Figure 1A) that were absent in a suspension containing Fe_2_O_3_ nanoparticles conjugated to FITC incubated aCSF for 48 h (Figure 1B). This suggests that Fe_2_O_3_ nanoparticles do not dissociate into ionic iron (Fe^3+^ ions) *in vivo* within 48 h of exposure to physiological conditions and this result is indicative of the stability of the nanoparticles prepared in this study. After ASV-30 was incubated with APTES-coated Fe_2_O_3_ nanoparticles for up to 5 h, a 50% reduction in the peak at 1,434 cm^−1^ (symmetric vibration of COO^−^ groups) was observed (Figure 2A), demonstrating that the COO^−^ groups of the ASV-30 molecules had reacted with the amine groups of the APTES coating to form new amide groups on the iron oxide surface. Concomitantly, an increase in the intensity of the band at 1,537 cm^−1^ (due to secondary N-H vibrations) was also observed, and this suggests the formation of new amide bonds as well. In order to understand the stability of the ASV-30 complex conjugated to the APTES-coated Fe_2_O_3_ nanoparticles, FT-IR spectroscopic studies were conducted. The FT-IR spectra of an APTES-coated Fe_2_O_3_ nanoparticles with ASV-30 complex and subsequently washed with aCSF was compared to a freshly prepared ASV-30 that was complexed with APTES-Fe_2_O_3_ nanoparticle for 3 h. As shown in Figure 2B, the aCSF washed ASV-30-APTES-Fe_2_O_3_ nanoparticles show characteristic peaks indicating that they are fairly stable in physiological aCSF.

### Distribution of systemically administered nanoparticles in the brain

**Figure 3:**
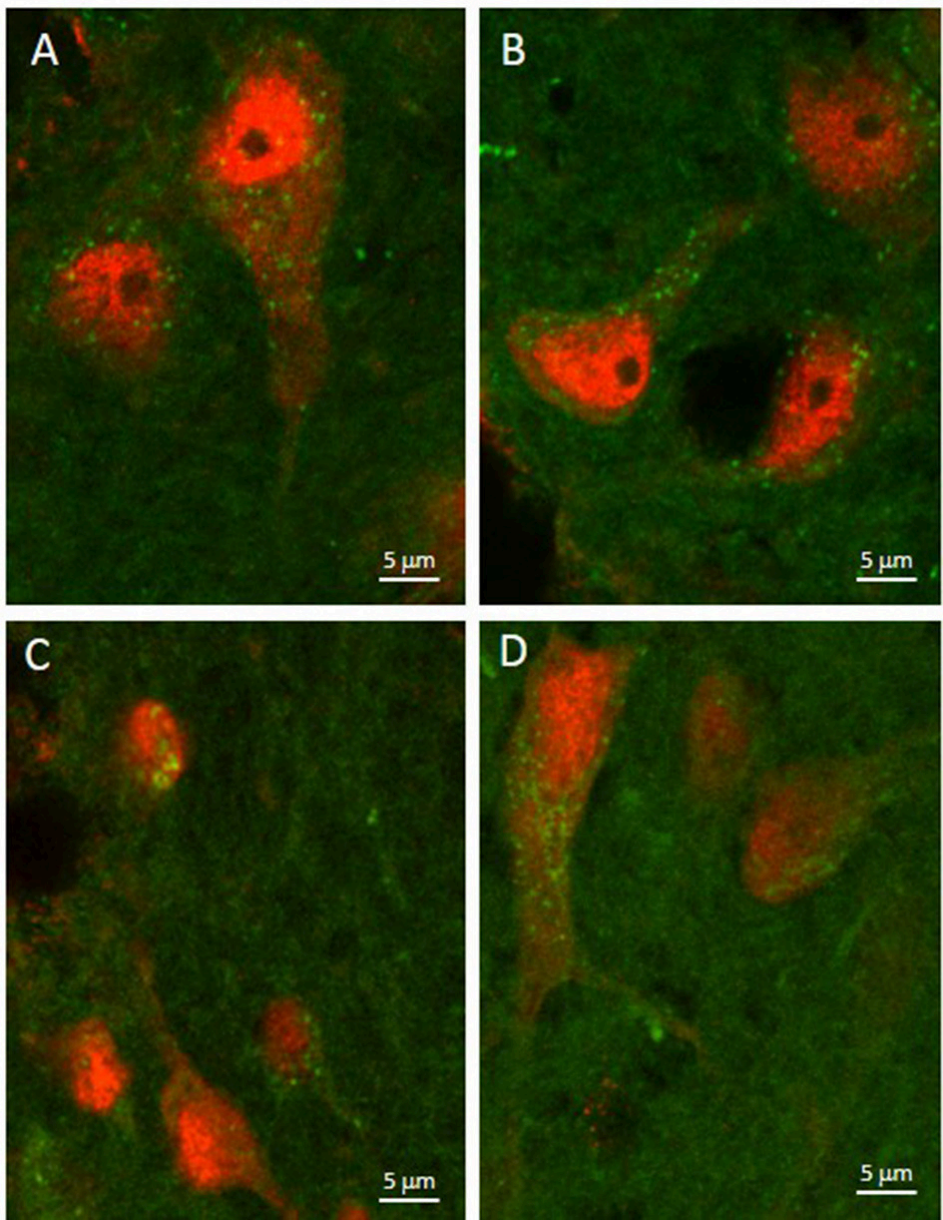
Iron oxide nanoparticle localization in the dorsal raphe nucleus and lateral septum. APTES-coated iron oxide nanoparticles effectively cross the blood brain barrier within 30 min of ip. administration regardless of ASV-30 conjugation (**A,C** = 87.7 μg/kg Fe_2_O_3_; **B**,**D** = 87.7 μg/kg Fe_2_O_3_ with 200 μg/kg ASV-30) in the **(A,B)** dorsal raphe nucleus and **(C,D)** lateral septum. Iron oxide nanoparticles are shown in green, while NeuN staining is shown in red.

**Figure 4:**
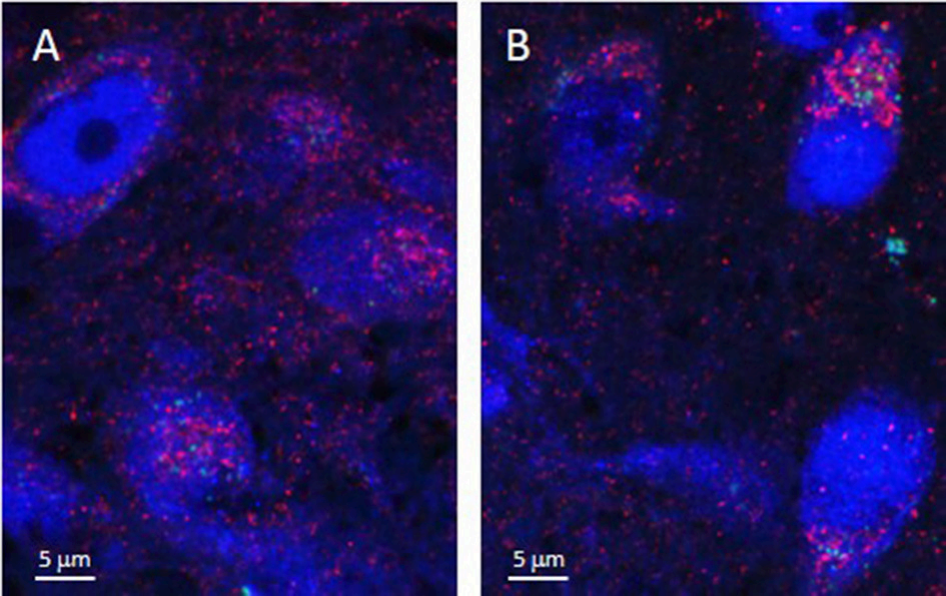
Iron oxide nanoparticle distribution in the dorsal raphe nucleus and lateral septum in relation to CRF_2_ receptor expression. APTES-coated iron oxide nanoparticles conjugated to ASV-30 (87.7 μg/kg Fe_2_O_3_ with 200 μg/kg ASV-30, ip) are localized with neurons expressing CRF_2_ receptors in the **(A)** dorsal raphe nucleus and **(B)** and lateral septum. Iron oxide nanoparticles are shown in green, NeuN staining is blue, and CRF_2_ receptor staining is red.

**Figure 5:**
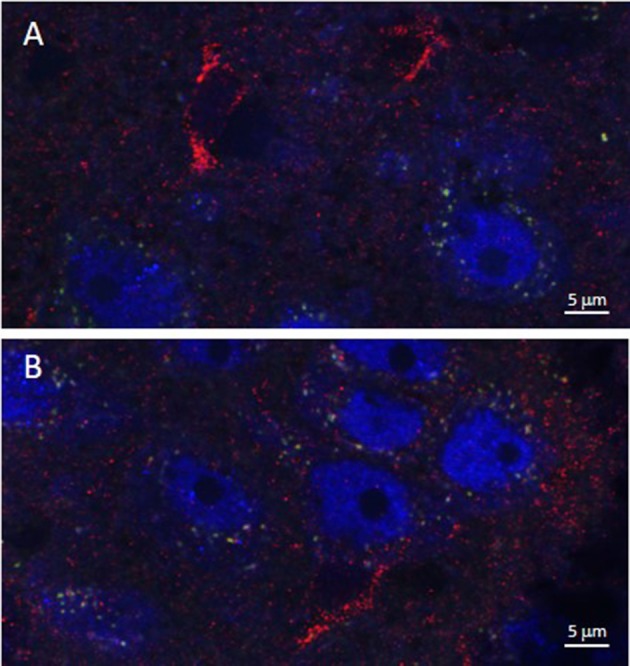
Iron oxide nanoparticle distribution in the dorsal raphe nucleus and lateral septum in relation to ferritin expression. APTES-coated iron oxide nanoparticles conjugated to ASV-30 (87.7 μg/kg Fe_2_O_3_ with 200 μg/kg ASV-30, ip) are not localized with ferritin in the **(A)** dorsal raphe nucleus and **(B)** and lateral septum. Iron oxide nanoparticles are shown in green, NeuN staining is blue, and ferritin staining is red.

As illustrated by Figure 3, APTES-coated Fe_2_O_3_ nanoparticles conjugated to ASV-30 effectively crossed the BBB. No apparent differences in the distribution of nanoparticles were observed between tissue obtained from rats treated with iron oxide compared to iron oxide+ASV-30 in regions of the brain associated with anxiety states, including the dRN and lateral septum (Figure 3). Iron oxide nanoparticles were widely distributed throughout the brain and were not found in greater concentrations in any particular region qualitatively compared to another. Furthermore, iron oxide nanoparticles+ASV-30 were localized with CRF_2_ receptor expressing neurons in the dRN and lateral septum (Figure 4). In contrast, iron oxide nanoparticles+ASV-30 showed little localization with ferritin in the dRN or lateral septum (Figure 5). Ferritin labeling was restricted to non-neuronal structures, likely glial cells (Figure 5, Moos and Morgan, 2004). Similar observations were found throughout the brain when sections were visualized at 0.1 μm intervals in the anterioposterior plane between the prefrontal cortex and cerebellum.

### Anxiolytic effects of iron oxide+ASV-30 nanoparticles

**Figure 6:**
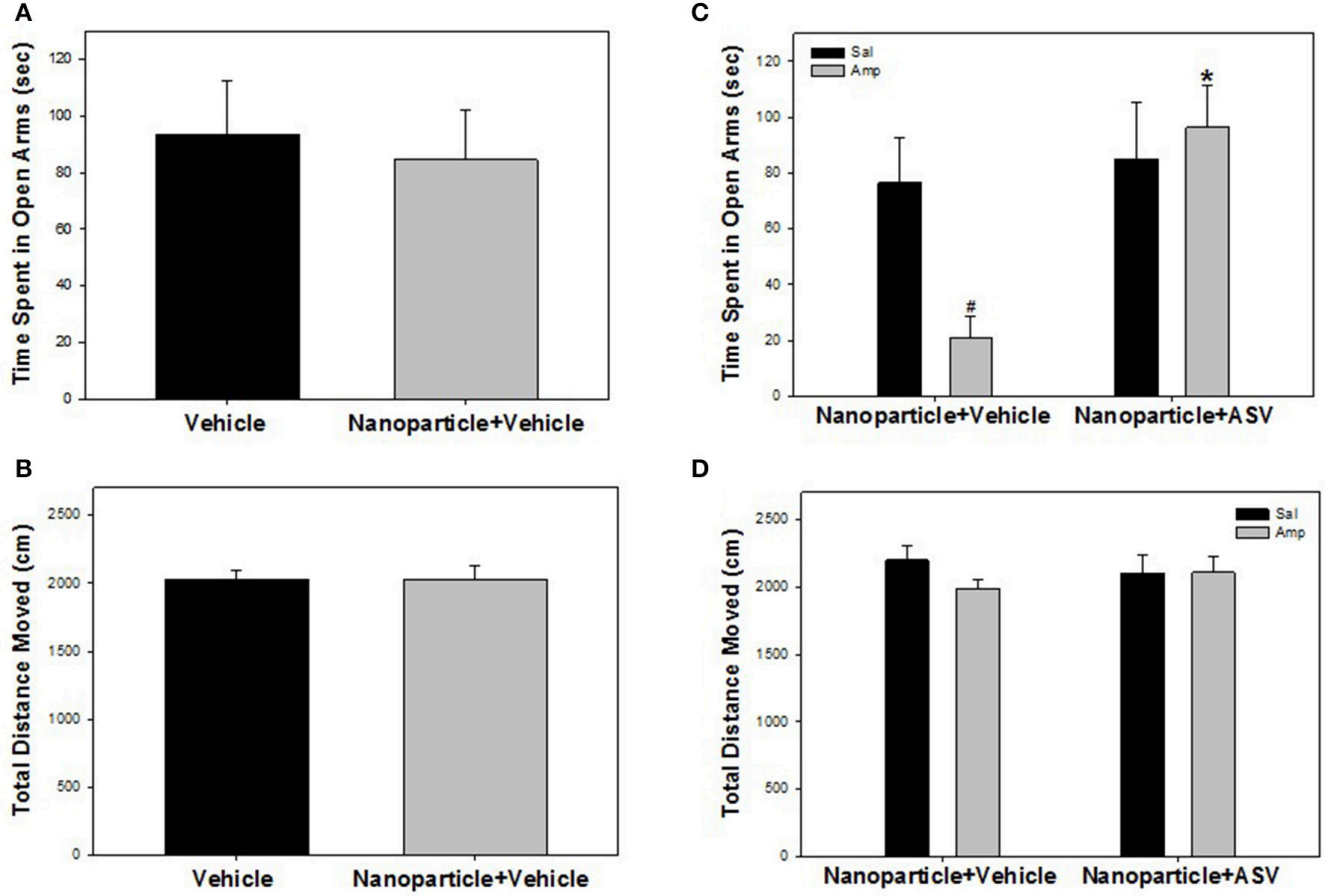
Iron oxide ASV-30 delivered as nanoparticle cargo is anxiolytic but does not affect locomotion. **(A)** Time spent in open arms of the elevated plus maze (EPM) did not differ between rats injected with vehicle and with APTES-coated iron oxide nanoparticles (87.7 μg/kg Fe_2_O_3_; ip) 30 min prior to testing. **(B)** APTES-coated iron oxide nanoparticle injections (87.7 μg/kg Fe_2_O_3_; ip) did not affect total locomotion in the EPM compared to vehicle controls. **(C)** Amphetamine pretreated rats (Amp) spend less time in the open arms of the elevated plus maze compared to saline pretreated rats (Sal) when both groups receive APTES-coated iron oxide nanoparticle+vehicle (87.7 μg/kg Fe_2_O_3_; ip) treatment 30 min prior to testing. The effects of amphetamine were reversed when APTES-coated iron oxide nanoparticle+ASV-30 (87.7 μg/kg Fe_2_O_3_ with 200 μg/kg ASV-30, ip) was administered 30 min prior to testing. **(D)** Neither amphetamine pretreatment or iron oxide nanoparticle administration affected locomotion in the EPM. ^#^Significant difference (*p* < 0.05) compared to saline pretreated nanoparticle+vehicle group. ^*^Significant difference (*p* < 0.05) compared to amphetamine pretreated nanoparticle+vehicle group.

Unconjugated APTES-coated Fe_2_O_3_ nanoparticle administration had no effect on anxiety-like behaviors or locomotion in the EPM (Figures 6A,B). There was no significant difference between vehicle- and nanoparticle-treated rats in time spent in the open arms of the EPM [*F*_(1, 20)_ = 0.108, *P* = 0.746] (Figure 6A). Furthermore, there was no significant difference in total distance moved between the treatment groups [*F*_(1, 20)_ < 0.001, *P* = 0.996; Figure 6B].

Increased anxiety-like behavior in amphetamine pretreated rats was reversed by ASV-30 delivered as nanoparticle cargo, without affecting locomotion in the EPM (Figures 6C,D). There was a significant interaction between pretreatment and treatment [*F*_(1, 48)_ = 4.660, *P* = 0.036] for time spent in open arms of the EPM. Rats pretreated with amphetamine and treated with iron oxide+vehicle spent less time in open arms than rats pretreated with saline and treated with iron oxide+vehicle (*P* = 0.014) indicating heightened anxiety-like behavior during amphetamine withdrawal (Figure 6C). However, amphetamine-pretreated rats receiving iron oxide+ASV-30 showed comparable time in open arms as compared to saline-pretreated rats receiving iron oxide+ASV-30 (*P* = 0.614) and exhibited greater time in open arms compared to amphetamine-pretreated rats receiving iron oxide+vehicle (*P* = 0.001; Figure 6C). Nanoparticle-ASV-30 treatment did not affect time spent in open arms for saline-pretreated animals (*P* = 0.692; Figure 6C). There was no significant effect of pretreatment [*F*_(1, 48)_ = 0.831, *P* = 0.367] or treatment [*F*_(1, 48)_ = 0.009, *P* = 0.923], and no significant interaction was found between pretreatment and treatment [*F*_(1, 48)_ = 0.954, *P* = 0.334] for distance moved in the EPM (Figure 6D).

## Discussion

APTES-coated Fe_2_O_3_ nanoparticles conjugated to ASV-30 were stable for at least 3 h in physiological conditions *in vitro*, and were observed throughout the brain 30 min following systemic administration *in vivo*. Thus, iron oxide nanoparticles were able to effectively cross the BBB without interference from conjugated ASV-30 in a time course where the peptide was still attached to the nanoparticle. The observation that iron oxide nanoparticles with ASV-30 were present in the brain within 30 min of systemic administration is similar timing observed for other nanoparticle delivery systems (Reimold et al., 2008; Ulbrich et al., 2009; Zensi et al., 2009).

Furthermore, iron oxide nanoparticles with ASV-30 were associated with neurons expressing CRF_2_ receptors in the dRN and lateral septum, both brain regions in which CRF_2_ receptors have been associated with increased anxiety (Henry et al., 2006; Vuong et al., 2010; Bledsoe et al., 2011). There was little co-localization between the Fe_2_O_3_+ASV-30 complex and the CRF_2_ receptor labeling. It is possible that this was a result of the Fe_2_O_3_+ASV-30 complex being taken up into CRF_2_-containing cells. We are unaware of a transport mechanism that would allow these complexes to be up-taken into neurons, since any iron-related transport mechanism would require binding of ferric (Fe^3+^) iron (Moos et al., 2007) and thus the complex would have dissociated. Another possibility is that the majority of CRF_2_ receptor antibody binding is intracellular and thus the binding site of the CRF_2_ antibody is at different cellular location when compared to the Fe_2_O_3_+ASV-30 complex that presumably bound to the extracellular domain of the CRF_2_ receptor. Iron oxide+ASV-30 nanoparticles were also localized near or with neurons that did not express CRF_2_ receptors and to a smaller degree, with non-neuronal cells. Therefore, the distribution of nanoparticles were not specific to CRF_2_-expressing neurons, but did appear to be preferentially localized to cells rather than significant accumulation in extracellular space. This is consistent with the distribution noted for poly(n-butylcyano-acrylate) nanoparticles for example, which were observed to be distributed throughout the brain tissue and localized to neuronal and non-neuronal cells but little distribution in the extracellular space (Reimold et al., 2008). Future work will need to determine non-neural distribution and effects of the Fe_2_O_3_+ASV-30 complex in organs such as the kidney and liver, and in peripheral tissues that highly express CRF_2_ receptor such as the heart, gastrointestinal tract, lungs, skeletal muscle, and vasculature (Bale and Vale, 2004).

The distribution of iron oxide+ASV-30 nanoparticles did not appear to overlap to any extent with expression of ferritin. Ferritin is a protein involved in iron storage and is typically not expressed in neurons but is observed in neural glia (Moos and Morgan, 2004). Ferritin thus has the potential to sequester iron-based nanoparticles, reducing bioavailability of transported cargo and increasing the probability of iron accumulation and neurotoxicity (Moos and Morgan, 2004; Hare et al., 2013). The current study thus suggests that Fe_2_O_3_ nanoparticles are not sequestered by ferritin in brain tissue. It is thought that ferric iron (Fe^3+^) is required for binding to ferritin (Moos and Morgan, 2004; Hare et al., 2013). Here we show that at physiological pH, iron oxide nanoparticles were stable for at least 48 h and did not dissociate into ionic iron. Therefore, our stable iron oxide nanoparticles do not undergo the characteristic redox reaction necessary to bind to ferritin and are unlikely to exhibit reduced bioavailability due to cellular sequestration. This finding does bring up the question of how Fe_2_O_3_ nanoparticles are eliminated by the brain. The major route of iron elimination in the brain is reabsorption to systemic circulation via CSF, but this method of transport requires ferric ion binding to proteins like ferritin or transferrin in the CSF for transport to blood (Moos et al., 2007). It is possible that Fe_2_O_3_ nanoparticles are small enough to diffuse from CSF to general circulation for elimination without the need for transport proteins, which needs to be tested in future work.

Furthermore, the immunohistochemical and behavioral assay employed here suggested sufficient neurobioavailability of ASV-30 as delivered by Fe_2_O_3_ nanoparticles. Anxiety-like behavior of rats undergoing amphetamine withdrawal was reduced to control levels by systemic administration of iron oxide+ASV-30. This finding is identical to that observed when ASV-30 is directly infused into the dRN or into the lateral ventricles of rats during amphetamine withdrawal (Vuong et al., 2010; Reinbold et al., 2014). Similarly, direct injection of ASV-30 into the dRN reduces anxiety-like behaviors in other rat models of heightened anxiety (Bledsoe et al., 2011). Importantly, iron oxide nanoparticle administration alone, or iron oxide+ASV-30 treatment had no effect on anxiety-like behavior of control animals and also did not affect general locomotion. Typically we and others have found that pharmacological blockade of CRF_2_ receptors in the brain has no effect on the anxiety-like behavior of control rats (e.g., saline pre-treated or non-manipulated rats; Takahashi, 2001; Vuong et al., 2010; Bledsoe et al., 2011; Reinbold et al., 2014). However, an exception to this was the finding that a CRF_2_ receptor antagonist infused into the ventricles increased anxiety-like behavior of saline-pretreated rats but this effect was not apparent in rats that did not receive any pretreatment (Reinbold et al., 2014). Reinbold et al. show that the saline pre-treatment in that particular study resulted in alterations to the ratio of CRF_1_ to CRF_2_ receptors in the lateral septum that may have resulted in CRF_2_ receptor antagonism being anxiogenic in those rats. Together, current findings suggest a specific effect of systemically administered, nanoparticle-conjugated ASV-30 on heightened anxiety-like behavior in rats that mimics findings from intracranial infusions in the majority of studies. Future work should assess the concentration of ASV-30 that reaches the brain via this method of administration, and also test the utility of APTES-coated Fe_2_O_3_ nanoparticles for the delivery of other peptides to the brain. Furthermore, testing the ability of systemically-administered Fe_2_O_3_+ASV-30 to produce anxiolytic effects in different animal models, and with other tests of anxiety-like behaviors, and determining whether these effects are long-lasting are all necessary for understanding the generalizability of this complex as a potential anxiolytic.

A second major consideration for the future is determining any long-term cellular effects of the APTES-coated Fe_2_O_3_ nanoparticles described here. Small diameter uncoated Fe_2_O_3_ nanoparticles promote biofilm formation and bacterial growth *in vitro* (Borcherding et al., 2014), but is not known whether coated iron oxide nanoparticles as used here would have a similar effect *in vivo*. Dimercaptosuccinic acid (DSMA)-coated Fe_2_O_3_ nanoparticles reduce the ability of PC12 cells to respond to nerve growth factor and are pro-apoptotic in rat sciatic nerve (Pisanic et al., 2007; Kim et al., 2013). These previously observed neurotoxic effects are thought to be a result of the DSMA coating rather than effects of Fe_2_O_3_ alone. The treatment of nanoparticles with DSMA prevents nanoparticle aggregation and thus is used to improve cellular uptake, but the resultant increase in iron accumulation appear to increase generation of reactive oxygen species (ROS) by Fe^2+^ (Moos and Morgan, 2004; Pisanic et al., 2007; Shubayev et al., 2009; Kim et al., 2013). The stability of the DSMA-free Fe_2_O_3_ nanoparticles in physiological pH described here suggests that formation of ROS in the brain is unlikely in response to nanoparticle administration in the current study. Furthermore, aminosilane coating (such as APTES) of Fe_2_O_4_ iron oxide nanoparticles does not affect cortical cell viability, which stands in contrast to other coatings such as poly-dimethylamine-co-epichlorhydrin-co-ethylendiamine (PEA) that induced neurotoxicity (Rivet et al., 2012). While APTES-coated Fe_2_O_3_ nanoparticles utilized here appear to have little reported toxicity, the *in vivo* cellular effects of these nanoparticles, including their ability to induce ROS, remains to be tested.

In conclusion, the current findings show that APTES-coated Fe_2_O_3_ nanoparticles represent a feasible delivery mechanism to transport a CRF-related peptide to the brain. Future work should focus on the generalizability of these findings to other peptides and should also determine the safety of chronic use of this nanomaterial. Further validation of the Fe_2_O_3_ nanoparticles described here as a stable system for delivery of peptide-based drugs to the brain has important implications for the study and treatment of a variety of neurological and psychiatric conditions where peptidergic systems are implicated.

## Author contributions

GF, RK, and NV designed the research; NV, JS, CW, TK, and GF wrote the manuscript and performed the research; CW, TK, and RK contributed reagents; CW, RK, NV, and GF analyzed data; and all authors contributed to the editing of the manuscript.

### Conflict of interest statement

The authors declare that the research was conducted in the absence of any commercial or financial relationships that could be construed as a potential conflict of interest. The reviewer TS and handling Editor declared their shared affiliation.

## Abbreviations

aCSF: Artificial cerebrospinal fluid
APTES: 3-Aminopropyltriethoxysilane
ASV-30: Antisauvagine-30
BBB: Blood-brain barrier
CRF: Corticotropin-releasing factor
CRF_1_: Corticotropin-releasing factor type 1
CRF_2_: Corticotropin-releasing factor type 2
DGL: Dendrigraft poly-L-lysines
DSMA: Dimercaptosuccinic acid
dRN: Dorsal raphe nucleus
FITC: Fluorescein isothiocyanate
FT-IR: Fourier Transform infrared spectroscopy
PEA: Poly-dimethylamine-co-epichlorhydrine-co-ethylendiamine
PEG: Polyethylene glycol
ROS: Reactive oxygen species
TGA: Thermogravimetric Analysis.
